# Batch fabrication of ultra-sharp atomic force microscope probes with stair-shaped handles for high-precision imaging

**DOI:** 10.1038/s41378-025-00986-4

**Published:** 2025-10-21

**Authors:** Aixi Pan, Xiaoli Zhu, Chenxu Zhu, Jian Yin, Md Soyaeb Hasan, Zhongyi Liu, Dayan Ban, Bo Cui

**Affiliations:** 1 Department of Electrical and Computer Engineering, Waterloo Institute for Nanotechnology, University of Waterloo, Waterloo, ON, N2L 3G1 Canada; 2 Department of Chemical Engineering, Waterloo Institute for Nanotechnology, University of Waterloo, Waterloo, ON, N2L 3G1 Canada; 3 School of Electronic Science and Technology, Eastern Institute of Technology, Ningbo, Zhejiang 315200 China

**Keywords:** NEMS, Electrical and electronic engineering

## Abstract

Atomic force microscope (AFM) systems rely on silicon (Si) probes for precise nanoscale characterization across diverse environments. However, fabricating high-aspect-ratio (HAR) and sharp Si tips and optimizing the handle geometries remain significant challenges. Conventional HAR probe fabrication methods lack scalability, precision, and cost efficiency, while cuboid-shaped handles risk obstructing laser detection and limiting compatibility. This study presents an innovative batch-fabrication strategy for high-performance Si AFM probes that integrate ultra-sharp HAR tips, rectangular cantilevers, and universally compatible stair-shaped handles. Notably, the tip fabrication process employs only low-cost microscale ultraviolet (UV) lithography, while still achieving nanoscale structural resolution. The fabricated probes exhibit a tip apex radius of 5 nm and a half-cone angle of 7.5°, enabling high-resolution and high-fidelity imaging. The novel stair-shaped handle geometry is introduced and fabricated via a single-step dry etching process, which provides unobstructed laser detection and ensures compatibility with a broad range of commercial AFM platforms. Durability testing demonstrates stable scanning performance for up to 8 hours within the 100 nm precision range, confirming the mechanical reliability of the design. This scalable, reproducible, and high-yield fabrication strategy represents a significant advancement in HAR AFM probe development, providing enhanced performance and extended applicability for diverse nanoscale imaging applications.

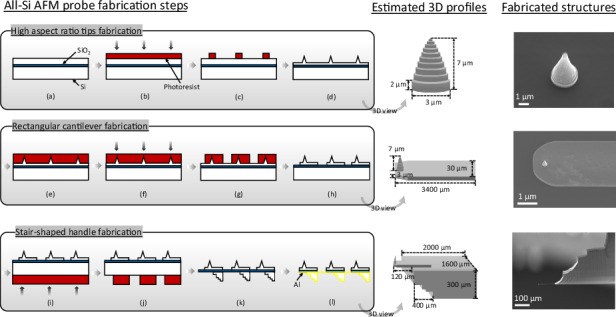

## Introduction

The AFM system has undergone a remarkable evolution, transitioning from a basic technique for surface morphology analysis into a multifaceted platform enabling nanomechanical testing^1,2^, characterization of magnetic and electric properties^3,4^, nanolithography^5–7^, and biological investigations^8–10^. At the heart of AFM’s operation is micro-cantilever force detection, a mechanism that offers distinct advantages over other scanning probe microscope (SPM) techniques: minimal sample preparation, no requirement for specialized treatments, and negligible risk of sample damage. Si has become a vital material in tapping mode imaging application^11,12^, widely favored for its use in tips, cantilevers and handles due to its excellent surface machinability and balanced elastic modulus. Si tips are generally easier to fabricate for tip structures with sharper apexes and smaller cone angles than those made from materials such as silicon nitride (Si_3_N_4_)^13,14^ or diamond^15^. For the cantilever, the unique mechanical and processing properties of Si allow for precise control over dimensional parameters, enabling accurate tuning of the spring constant and resonant frequency. These advantages distinguish Si from conventional materials such as Si_3_N_4_^16^ and polymer^17^ in fabricating high-performance HAR AFM probes. Additionally, its superior processability facilitates nanoscale fabrication of the handle^18,19^ into customized shapes, significantly enhancing its versatility and suitability for advanced AFM applications.

Currently, two types of Si AFM probes are suitable for large-scale manufacturing. The first is the in-plane AFM probe, where the tip lies co-planar with the handle. This configuration enables the scalable fabrication of rigid, complementary metal-oxide-semiconductor (CMOS)-compatible structures using standard microelectromechanical systems (MEMS) processes at relatively low cost^14,20–23^. However, in-plane probes still exhibit limited sensitivity and dynamic performance compared to conventional out-of-plane cantilever designs, and their non-standard geometry and mounting configuration limit compatibility with most commercial AFM systems. The complexity of the integrated structure also leads to higher fabrication costs, while the difficulty of frequent replacement after tip degradation further constrains their practical applicability. The second category comprises the out-of-plane AFM probe, which is standard in commercial systems. Probes fabricated by companies like NanoWorld^24^ (see Fig. SI-1) typically feature a pyramidal tip with a radius of ~10 nm and a half-cone angle ranging from 30° to 70°, supported by a rectangular cantilever and a cuboid handle. These probes are batch-fabricated using cost-effective wet etching techniques, commonly involving potassium hydroxide (KOH) or tetramethylammonium hydroxide (TMAH)^25,26^. While this approach offers reduced costs, it inherently produces broad tip angles and limited aspect ratios, which restrict the probe’s ability to access deep or narrow features.

HAR AFM tips are essential for probing deep or confined nanostructures, but current fabrication techniques fall short in achieving both precision and scalability. Conventional methods such as focused ion beam (FIB) milling, electron/ion beam-induced deposition (EBID/IBID), carbon nanotube (CNT) attachment, and the gallium (Ga)-silver (Ag) crystallization technique each have notable limitations. FIB^12,15,25^ milling provides nanometer-scale precision by sputtering material with a focused gallium ion (Ga⁺) beam, but its slow tip-by-tip processing nature and high costs make it unsuitable for large-scale production. EBID/IBID methods can achieve high aspect ratios exceeding 1000:1^27,28^, but they are also expensive and time-consuming. CNT-based tips offer exceptional apex sharpness and extended pillar heights, but poor control over alignment, positioning, and length during attachment limits their reproducibility and scalability^29^. The Ga-Ag crystallization method, proposed by Yazdanpanah et al. ^30^, can produce ultralong needles at room temperature through spontaneous alloy crystallization. However, the process lacks control over tip geometry and is not compatible with volume manufacturing. Given these limitations, there remains an urgent need for a cost-effective, scalable, and technically robust approach to HAR Si tip fabrication.

Apparently, the probe handle must not block the incident or reflected laser beam, so commercially available handles typically feature a 54.7-degree slope resulting from the anisotropic KOH or TMAH etching of the silicon handle. But for our process, we utilized dry etching, instead of KOH or TMAH wet etching, to etch the handle, to obtain a vertical sidewall of the handle that makes the tip handling by tweezers much easier than the 54.7° sloped sidewall. However, the vertical sidewall next to the cantilever might block the laser path for some AFM tools. To address this, handles can be designed with “stair-like” profiles that mimic the sloped profile by wet etching.

In this work, we developed a novel fabrication strategy for the Si AFM HAR probe with an innovative stair-shaped handle design. This strategy integrates a non-switching pseudo-Bosch process using SF_6_/C_4_F_8_ gases with periodic O_2_ plasma shrinking of resist mask structure for tip etching^31^, while the stair-shaped handle is etched by utilizing the reactive ion etching (RIE) micro-loading effect^18,32,33^ and RIE-lag^19,34,35^, which is also often termed as aspect-ratio dependent etching (ARDE, higher aspect ratio gives slower etching) effect in the literature^36^. We demonstrated that the final tip apex diameter can reach under 10 nm with a half-cone angle of 7.5°. The resulting probes are compatible with various AFM models without laser blocking, ensuring accurate, high-resolution scanning results. Furthermore, these probes demonstrated a remarkable durability of up to 8 hours under 100 nm precision conditions, making them a reliable solution for advanced AFM applications.

## Simulation and experiment

### Simulation and design

**Fig. 1:**
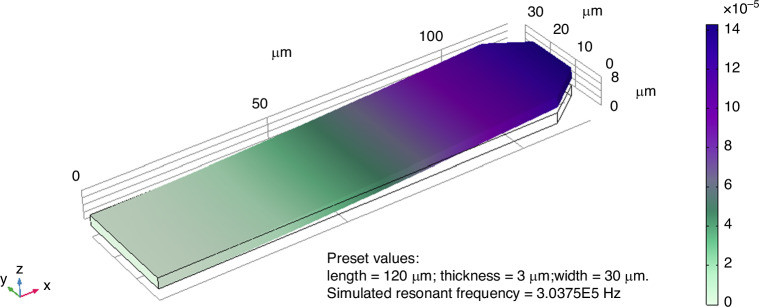
The resonant frequency and spring constant were simulated for a rectangular cantilever using COMSOL Multiphysics with dimensions of 120 μm in length, 30 μm in width, and 3 μm in thickness

The performance of Si probes is fundamentally influenced by the cantilever’s mechanical properties, including the spring constant and resonant frequency. The spring constant, representing cantilever stiffness, depends on the material’s Young’s modulus and the cantilever’s dimensions- width, thickness, and length. Specifically, it increases with the width and thickness but decreases as the length grows. The resonant frequency, which directly dictates scanning speed and resolution, is influenced by the spring constant and the material density. It is directly proportional to the thickness and inversely proportional to the square of the length. These relationships highlight the critical need for precise dimensional tuning to achieve the desired mechanical behavior. For tapping-mode AFM, balancing stiffness and resonant frequency is essential, with fabrication constraints further guiding the dimension selection. Based on the analysis in Fig. SI-3, a set of dimensions of 120 μm in length, 30 μm in width, and 3 μm in thickness was designed using MATLAB. A geometric model of the cantilever was constructed in COMSOL Multiphysics, featuring a rectangular shape with a small trapezoid at the free end (Fig. 1). Simulations revealed elastic deformation under applied forces, with deflection proportional to the force (Fig. SI-4). A maximum force of 50.62 μN resulted in a displacement of 2.52 μm, while a minimum force of 9.38 μN produced a displacement of 0.42 μm (note that tapping mode AFM operation often has a displacement of tens of nm). The simulated spring constant was thus 19.64 N/m, closely aligning with MATLAB results. Vibrational response analysis further indicated a resonant frequency of 303.75 kHz, as shown in Fig. 1.

### Fabrication process

**Fig. 2:**
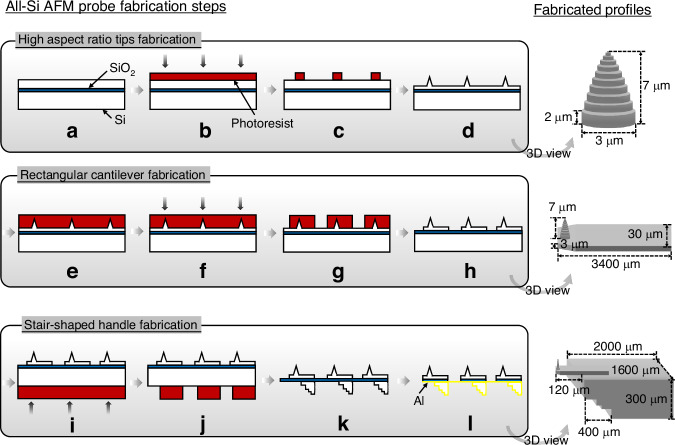
The schematic profiles and the fabrication process of the all-Si AFM probe: **a** wafer cleaning and HMDS treatment, **b** resist coating and lithography for tip patterning, **c** resist development, **d** Si tip etching via Bosch and pseudo-Bosch etching, **e** resist patterning to protect tips, **f** lithography for cantilever patterning, **g** resist development, **h** dry-etching cantilever patterns into the device layer, **i** resist coating and lithography for handle patterning, **j** resist development, **k** deep Si etching to the BOX stop layer, **l** Al deposition on cantilever backside for reflectivity

The fabrication of the Si AFM probe in this work involves three primary components, including the HAR tip, the rectangular cantilever, and the stair-shaped handle. As illustrated in Fig. 2, the proposed fabrication process has been performed using a Si-on-insulator (SOI) wafer. The SOI wafer consists of a 10 µm-thick device layer, a 2 µm buried oxide (BOX) layer, and a 400 µm handle layer. The process begins with rinsing the wafer with acetone, isopropanol (IPA), and a 2-minute oxygen (O_2_) plasma treatment with 20 W radio frequency (RF) power, 20 sccm O_2_ gas flow, and 20 mTorr chamber pressure. After cleaning, hexamethyldisilane (HMDS) treatment is applied to improve adhesion between the photoresist and the substrate. HMDS bonds with the -OH groups on the Si surface, leaving a hydrophobic methyl layer that prevents aqueous developer intrusion during resist development, thereby minimizing resist peeling (Fig. 2a).

For the HAR tip fabrication, a 3.3 µm-thick negative photoresist (AZ nLof 2035) is spin-coated and pre-baked at 110°C for 1 minute. Using a direct write UV lithography system (MLA150, Heidelberg Instruments), 3.0 µm-diameter pillars are patterned to define the tip structure along with semi-enclosed protective Si structures (Fig. 2b). After post-exposure baking (PEB) at 110°C for 1 minute and development (Fig. 2c), the tip pattern is transferred into the Si layer using a combination of the Bosch process and a pseudo-Bosch process with periodic O_2_ plasma shrinking (Fig. 2d). This sequence involves four Bosch cycles followed by 35 cycles of modified resist shrinkage etching, achieving a tip height of 7 µm.

Following tip profile formation, the remaining resist is removed with a 2-minute O_2_ plasma. A 10 µm-thick AZ4620 resist layer is then spin-coated to protect the fabricated tip structures (Fig. 2e). This resist is pre-baked at 95°C for 90 seconds, exposed using a 405 nm MLA system with a dose of 900 mJ/cm^2^ and developed (Fig. 2f, g). The cantilever pattern is subsequently etched into the device layer using 6 Bosch cycles, achieving a depth of 3 µm (Fig. 2h).

To define the handle, another 10 µm AZ4620 resist layer is spin-coated and patterned as an etching mask on the backside of the SOI wafer (Fig. 2I, j). The handle structures are etched into the Si substrate using the Bosch process to a depth of 320 µm. After removing the remaining resist, isotropic Si etching is performed with an SF_6_ gas recipe (160 sccm SF_6_, 20 W RF power, 1000 W inductively coupled plasma (ICP) power, and 15 mTorr pressure for 20 minutes) to remove lateral Si walls. The etching process continues using the standard Bosch until the BOX layer is reached (Fig. 2k). Finally, the BOX layer is removed using buffered oxide etch (BOE). To enhance optical reflectivity for the AFM imaging system, a thin layer of aluminum (Al) is deposited on the backside of the cantilevers (Fig. 2l).

## Results and discussion

### Si tip and cantilever fabrication

**Fig. 3:**
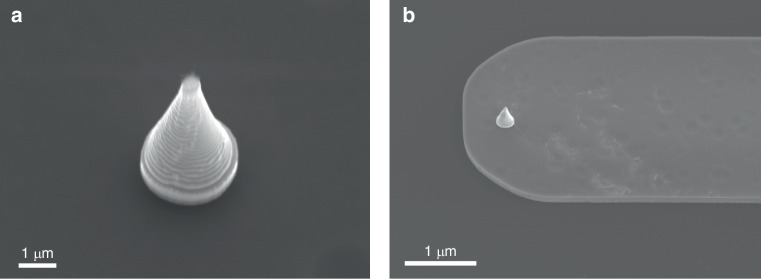
SEM images of (**a**) the etched tip structure, **b** the etched tip and cantilever. All images were captured using a JOEL JSM-7200F SEM with the samples on a 20° tilted stage

Within the overall sample area of 5 cm × 5 cm (Fig. SI-5), the fabrication process begins on the device layer of the SOI wafer by etching a Si tip using a 3 μm diameter mask. The objective aims to fabricate a 7 μm-tall Si tip with a sharp apex for high-resolution scanning and a sturdy cylindrical base for mechanical stability, as detailed in our previous work^31^. The core advantage of this process is to fabricate nanoscale structures solely through microscale UV exposure, and this approach involves two critical steps: first, the standard Bosch is employed to etch a 2 μm cylindrical base. Next, a non-switching pseudo-Bosch process using SF_6_/C_4_F_8_ gases and periodic O_2_ plasma shrinking of the resist mask creates the tapered upper portion. Figure 3a displays the etched Si tip with an etching height of 7 µm, revealing a tapered structure as expected. The base diameter of the tip is 3 µm, tapering to a sharp apex of 200 nm. The sample in Fig. 3 is tilted at 20°, causing the tip to appear less sharp compared to its final appearance when mounted on a 90° vertical stub. After fabricating the tip structures, a thick layer of photoresist was applied to protect them during the subsequent cantilever etching process. The cantilever design, based on simulation results, featured a width of 30 µm, a length of 120 µm, and a thickness of 3 µm, determined by subtracting the tip height (7 µm) from the 10 µm Si device layer of the SOI wafer. Etching down to the buried oxide layer produced the cantilever and tip structures, as shown in Fig. 3b.

### Stair-shaped handle fabrication

After completing the front-side fabrication on the SOI wafer, attention shifted to the challenges of fabricating the AFM handle. To fabricate the “stairs” with a single lithography plus etching step (instead of one lithography/etching for each stair), the consideration of the micro-loading effect^37^ and RIE-lag is utilized for advantage. The micro-loading effect refers to the local depletion of reactive species in areas with high pattern density, resulting in a slower etch rate in those regions compared to more isolated features^18,32^. Similarly, RIE lag describes the reduced etching rate observed in narrow openings relative to wider ones, due to limited ion transport and aspect ratio–dependent etch dynamics^19^. As demonstrated by the etching test in Fig. SI-7, the combined influence of the loading effect and RIE lag results in etch depth variations that correlate with the mask opening size: smaller openings yield shallower trenches, whereas larger openings lead to increased etch depths under identical processing conditions.

**Fig. 4:**
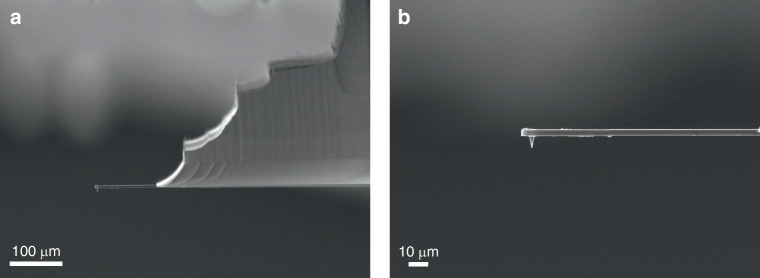
SEM images of (**a**) the side view of the AFM probe with the cantilever length of 120 µm, and **b** the enlarged view of the cantilever and the tip. All images were captured using a JOEL JSM-7200F SEM with the samples mounted on a 90° tilted stage

Based on these observations, the layout was optimized by incorporating a series of rectangular structures with progressively increasing feature sizes, thereby enhancing the depth contrast induced by local pattern density and opening geometry. Following this design optimization, the stair-shaped handle pattern was defined by resist spin-coating and alignment lithography. The handle structures were etched into the silicon substrate using the Bosch process until the deepest stair step reached a depth of 320 µm. After resist removal, isotropic silicon etching was performed using an SF_6_-based recipe to eliminate the lateral silicon walls. The etching process then resumed with the standard Bosch sequence until the buried BOX layer was reached. Finally, the BOX layer was removed using a BOE solution. A total of 60 probes were fabricated and inspected individually via SEM, among which 46 were found to be intact and functional, resulting in a yield of 77%. The probes featured a tip height of 7 µm, a cantilever thickness of 3 µm, and a handle thickness of 300 µm. The cantilever length was relatively uniform at approximately 120 µm (Table SI-1), with minor variations caused by substrate non-flatness and slight alignment errors during photolithography. Failure analysis revealed that most damaged cantilevers were located near the wafer corners (Table SI-1 and Fig. SI-6), attributed to insufficient edge margins in the mask design. Narrow boundaries increased the risk of mechanical damage during handling, so wider margins are recommended in future designs to improve robustness and yield. Representative SEM images of an intact probe with a tip diameter of approximately 200 nm are shown in Fig. 4a and b.

### Oxidation sharpening

**Fig. 5:**
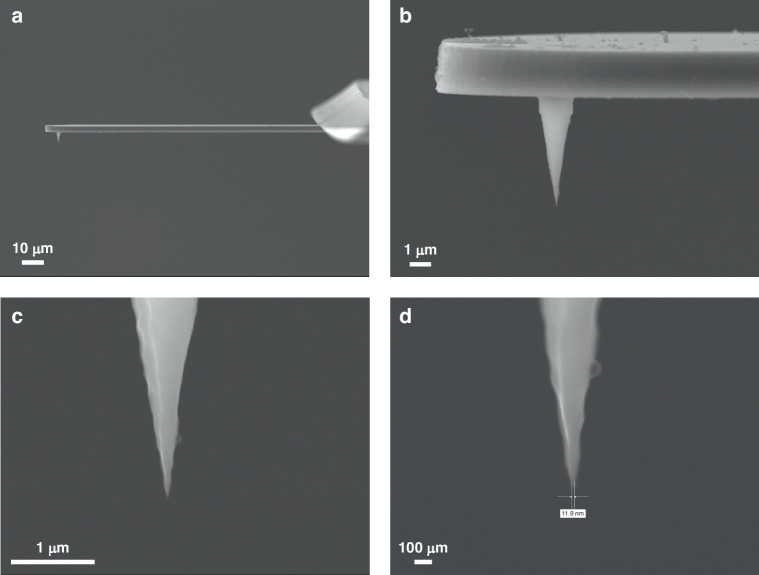
SEM images of (**a**) the final product of the AFM probe with 120 µm cantilever after oxidation sharpening, and **b**–**d** the enlarged view of the cantilever and the tip. All images were captured using a JOEL JSM-7200F SEM with the samples mounted on a 90° tilted stage

The SEM images reveal that the fabricated probes currently exhibit a tip diameter of 200 nm, necessitating further sharpening techniques. As reported in many studies^38–40^, thermal oxidation of three-dimensional structures is subject to slower oxidation rates at concave and convex angles due to stress-induced effects. At relatively low temperatures ( ~ 950°C), thermal stress plays a more significant role, while temperatures exceeding 1100°C tend to alleviate uneven oxidation. Given that the etched tip already possesses a diameter of 200 nm and a preliminary conical shape, thermal oxidation was conducted at 950°C for 8 hours. Following the removal of the thermally grown SiO_2_ layer using BOE, the final AFM probe (Fig. 5) exhibits a sharpened Si tip with an apex diameter of 10.54 ± 0.88 nm, as summarized in Table SI-1.

### Performance test on AFM system

**Fig. 6:**
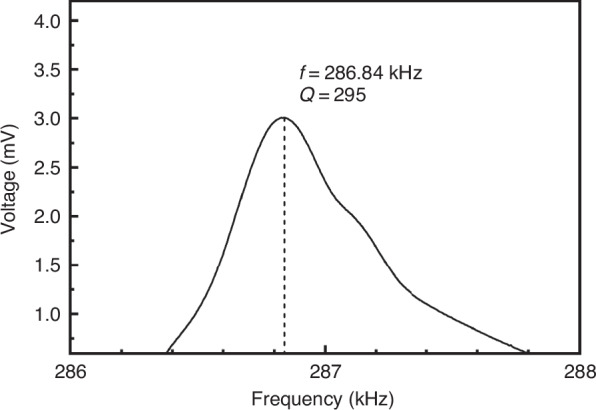
Frequency response at resonance and Q-factor of a cantilever

The Si probe with a HAR tip and stair-shaped handle was mounted onto the FM-Nanoview1000 AFM system (Fisherman Company, Suzhou, China) for testing. The reflected laser signal was measured at 2.5 V immediately after the cantilever release. After depositing a 30 nm aluminum layer on the cantilever surface, the reflected signal increased to 3.8 V, indicating improved reflectivity. The probe exhibited a resonant frequency of 286.84 kHz (Fig. 6), a spring constant of 19 N/m, and a quality factor (Q-factor) of 295. The measured resonant frequency and spring constant differed by only 5.9% and 3.4% from the simulated values, respectively, confirming that the fabricated probe dimensions closely matched the design specifications. All AFM scans were conducted at a scanning rate of 0.6 Hz with a resolution of 256 pixels per line.

**Fig. 7:**
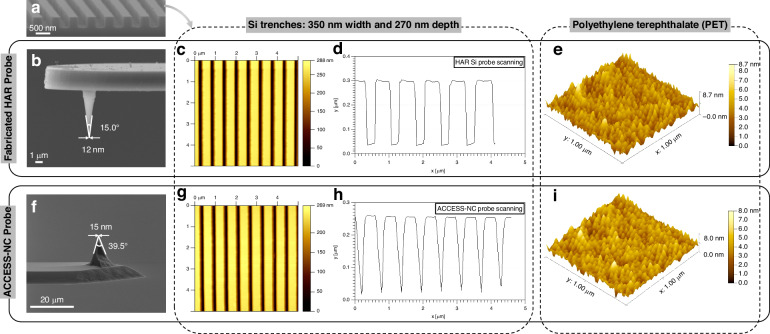
**a** SEM image of a Si sample with trenches measuring 350 nm in width and 270 nm in depth, **b** apex diameter and cone angle of the fabricated probe, **c**, **d** AFM scanning results obtained using the fabricated Si probe on Si trenches, **e** AFM scanning result obtained using the fabricated Si probe on a PET film, **f** apex diameter and cone angle of the ACCESS-NC probe, **g**, **h** AFM scanning result obtained using the ACCESS-NC probe on Si trenches, **i** AFM scanning result obtained using the ACCESS-NC probe on a PET film

To evaluate the probe’s aspect ratio performance, a nanoscale Si test sample with trenches measuring 350 nm in width and 270 nm in depth was prepared (Fig. 7a). Initial scanning was performed using the fabricated Si probe with a 120 µm cantilever (Fig. 7b). The results, shown in Fig. 7c and d, reveal that the measured trench profile aligns closely with the corresponding SEM image, validating the probe’s accuracy. For comparison, the sample was also scanned using an uncoated ACCESS-NC probe from AppNano company, whose tip apex measures approximately 15 nm with a half-cone angle of 20° (Fig. 7f). While this commercial probe successfully reached the trench bottoms, the resulting scans were inaccurate, as evidenced in Fig. 7g and h. The results highlight the exceptional performance of the batch-fabricated probes developed in this study. These probes not only demonstrate comparable capabilities to leading commercial alternatives when scanning standard samples but also surpass them in accurately capturing high aspect ratio features with enhanced precision.

A transparent optical-grade polyethylene terephthalate (PET) film, widely utilized in optical applications for its smooth and uniform surface, was selected to evaluate the tip apex sharpness. Due to the challenges of observing PET surface morphology using SEM, AFM was employed to demonstrate the probe’s capabilities. The ACCESS-NC tip, sharpened through conventional wet etching to approximately 15 nm, delivered comparable scanning results to the fabricated probe, as shown in Fig. 7e and i. However, the high aspect ratio probe developed in this study, featuring a sharper tip apex, captured finer surface details, resulting in noticeably enhanced imaging performance. This sub-10 nm scanning result further indicates that the actual apex radius of the fabricated probe must be smaller than 5 nm, highlighting a degree of measurement error in the SEM analysis. This finding underscores the superior sharpness and precision of the fabricated probe compared to the conventional probes.

Durability tests (Fig. SI-9) confirmed the robustness of the fabricated probes, demonstrating a scanning lifespan of at least 3 hours while maintaining a tip apex diameter below 40 nm, and up to 8 hours within the 100 nm precision range. Despite gradual tip wear over time, the image quality remained consistent throughout the entire scanning period. The primary wear mechanism is attributed to electrochemical corrosion, in which the progressive formation of a surface oxide layer on silicon accelerates tip degradation during prolonged operation. Notably, even after 8 hours of continuous use, the probe remained functional and can continue to be used for general surface imaging tasks. To assess repeatability and enhance the yield rate, the fabrication process was repeated for a second batch of all-Si probes (from Figs. SI-10 to SI-13). By testing on the Aist-NT SmartSPM 1000 in AFM tapping mode, we verified the versatility of the fabricated probe, further achieving an improved yield rate of 84%. As a result, the probes were shown to be compatible with various AFM models without obstructing the laser, and their suitability for tapping-mode imaging enabled effective resolution of structures with high aspect ratios and superior scanning resolution. After probe fabrication, tip coating can further expand the application range of silicon probes. For instance, a thin layer of gold can be applied for detecting covalent bonds in biological molecules and their interactions with surfaces^41^. Coating with diamond-like carbon (DLC) enhances the probe’s durability due to its exceptional hardness^42^. Additionally, magnetic films can be deposited to enable the investigation of magnetic properties in sample surfaces, supporting applications such as magnetic force microscopy (MFM)^43^.

## Summary and conclusions

This study successfully demonstrated the volume production of silicon atomic force microscope probes, featuring ultra-high aspect ratio tips fabricated via the non-switching pseudo-Bosch etching with O_2_ shrinking, silicon cantilevers, and non-laser blocking stair-shaped handles using the loading effect and reactive ion etching lag. Notably, the process employs only three cycles of cost-effective ultraviolet lithography and plasma etching to fabricate both the nanoscale high-aspect-ratio tip and the newly proposed universal handle, representing a significant advancement in comprehensive AFM probe fabrication. The resulting probes exhibit outstanding performance metrics, including a tip apex radius smaller than 5 nm, a half cone angle of 7.5°, a resonant frequency of 286.84 kHz, and a spring constant of 19 N/m. These parameters align closely with simulation predictions for cantilevers with a thickness of 3 µm and a length of 120 µm, underscoring the precision and reliability of this fabrication approach. The fabricated probe features a high aspect ratio, enabling efficient scanning of deep or tall structures, while its ultra-sharp tip provides exceptional scanning resolution. Additionally, the innovative, easy-to-handle stair-shaped design eliminates the risk of laser obstruction commonly associated with traditional cuboid handles. Furthermore, the simplified fabrication process, characterized by short production cycles and high yield, underscores the potential of these ultrasharp probes to significantly enhance the performance of atomic force microscopy systems and expand their applications across diverse scientific and industrial domains.

## Supplementary information

Supplementary Information

## References

[1] Han, B. et al. AFM-nanomechanical test: an interdisciplinary tool that links the understanding of cartilage and meniscus biomechanics, osteoarthritis degeneration, and tissue engineering. *ACS Biomater. Sci. Eng.* **3**, 2033–2049 (2017). 31423463 10.1021/acsbiomaterials.7b00307 PMC6697429

[2] Withers, J. R. & Aston, D. E. Nanomechanical measurements with AFM in the elastic limit. *Adv. Colloid Interf. Sci.* **120**, 57–67 (2006). 10.1016/j.cis.2006.03.002 16712762

[3] Gisbert, V. G., Amo, C. A., Jaafar, M., Asenjo, A. & Garcia, R. Quantitative mapping of magnetic properties at the nanoscale with bimodal AFM. *Nanoscale* **13**, 2026–2033 (2021). 33449980 10.1039/d0nr08662b

[4] Qi, Y. Investigation of organic films by atomic force microscopy: Structural, nanotribological and electrical properties. *Surf. Sci. Rep.* **66**, 379–393 (2011).

[5] Martín, C., Rius, G., Borrisé, X. & Pérez-Murano, F. Nanolithography on thin layers of PMMA using atomic force microscopy. *Nanotechnology* **16**, 1016–1022 (2005).

[6] Tang, X. & Lai, K. W. C. Substrate effect on atomic force microscopy-based nanolithography of graphene. *IEEE Trans. Nanotechnol.* **15**, 607–613 (2016).

[7] Zheng, J., Chen, Z. & Liu, Z. Atomic force microscopy-based nanolithography on silicon using colloidal Au nanoparticles as a nanooxidation mask. *Langmuir* **16**, 9673–9676 (2000).

[8] Uchihashi, T., Watanabe, H., Fukuda, S., Shibata, M. & Ando, T. Functional extension of high-speed AFM for wider biological applications. *Ultramicroscopy.* **160**, 182–196 (2016). 26521164 10.1016/j.ultramic.2015.10.017

[9] Jalili, N. & Laxminarayana, K. A review of atomic force microscopy imaging systems: Application to molecular metrology and biological sciences. *Mechatronics* **14**, 907–945 (2004).

[10] Alsteens, D. et al. High-resolution imaging of chemical and biological sites on living cells using peak force tapping atomic force microscopy. *Langmuir* **28**, 16738–16744 (2012). 23198968 10.1021/la303891j

[11] Ding, X. et al. A super high aspect ratio atomic force microscopy probe for accurate topography and surface tension measurement. *Sens. Actuators A Phys.* **347**, 113891 (2022).

[12] Samaan, M. et al. Fabrication of high aspect ratio atomic force microscope probes using focused ion beam milled etch mask. *Microelectron. Eng.* **267-268**, 111909 (2023).

[13] Kitazawa, M., Shiotani, K. & Toda, A. Batch fabrication of sharpened silicon nitride tips. *Jpn. J. Appl. Phys., Part 1: Regul. Pap. Short. Notes Rev. Pap.* **42**, 4844–4847 (2003).

[14] Geerlings, J. et al. Design and fabrication of in-plane AFM probes with sharp silicon nitride tips based on refilling of anisotropically etched silicon moulds. *J. Micromech. Microeng.* **24**, 105013 (2014).

[15] Gacka, E. et al. Focused ion beam-based microfabrication of boron-doped diamond single-crystal tip cantilevers for electrical and mechanical scanning probe microscopy. *Measurement (Lond)* **188**, 110373 (2022).

[16] Grow, R. J., Minne, S. C., Manalis, S. R. & Quate, C. F. Silicon nitride cantilevers with oxidation-sharpened silicon tips for atomic force microscopy. *J. Microelectromech. Syst.* **11**, 317–321 (2002).

[17] Yu, F. et al. Design, fabrication, and characterization of polymer-based cantilever probes for atomic force microscopes. *J. Vacuum Sci. Technol. B, Nanotechnol. Microelectron.: Mater. Process. Measurement Phenomena* **34**, 06KI01 (2016).

[18] Karttunen, J., Kiihamäki, J. & Franssila, S. Loading effects in deep silicon etching. In: Proceedings Micromachining and Microfabrication Process Technology VI. (2000). SPIE. 4174, 90–97 (2000).

[19] Jansen, H. et al. RIE lag in high aspect ratio trench etching of silicon. *Microelectron. Eng.* **35**, 45–50 (1997).

[20] Legrand, B. et al. Multi-MHz micro-electro-mechanical sensors for atomic force microscopy. *Ultramicroscopy* **175**, 46–57 (2017). 28110263 10.1016/j.ultramic.2017.01.005

[21] Ried, R. P., Mamin, H. J., Terris, B. D., Fan, L.-S. & Rugar, D. 6-MHz 2-N/m piezoresistive atomic-force-microscope cantilevers with INCISIVE Tips. *J. Microelectromech. Syst.* **6**, 294–302 (1997).

[22] Schwab, L. et al. Very-high-frequency probes for atomic force microscopy with silicon optomechanics. *Microsyst. Nanoeng.* **8**, 32 (2022). 35371536 10.1038/s41378-022-00364-4 PMC8931076

[23] Walter, B., Mairiaux, E. & Faucher, M. Atomic force microscope based on vertical silicon probes. *Appl Phys. Lett.* **110**, 243101 (2017).

[24] Russell, P. & Krause, O. *AFM Probe Manufacturing*. (NanoWorld Innovative Technologies, 2008).

[25] Knittel, P., Hibst, N., Mizaikoff, B., Strehle, S. & Kranz, C. Focused ion beam-assisted fabrication of soft high-aspect ratio silicon nanowire atomic force microscopy probes. *Ultramicroscopy* **179**, 24–32 (2017). 28384541 10.1016/j.ultramic.2017.03.031

[26] Resnik, D., Vrtacnik, D., Aljancic, U., Mozek, M. & Amon, S. Different aspect ratio pyramidal tips obtained by wet etching of (100) and (111) silicon. *Microelectron. J.* **34**, 591–593 (2003).

[27] Brown, J. et al. Electrically conducting, ultra-sharp, high aspect-ratio probes for AFM fabricated by electron-beam-induced deposition of platinum. *Ultramicroscopy* **133**, 62–66 (2013). 23770730 10.1016/j.ultramic.2013.05.005

[28] Nanda, G., van Veldhoven, E., Maas, D., Sadeghian, H. & Alkemade, P. F. A. Helium ion beam induced growth of hammerhead AFM probes. *J. Vac. Sci. Technol. B, Nanotechnol. Microelectron.: Mater., Process., Meas., Phenom.* **33**, 06F503 (2015).

[29] Engstrom, D. S. et al. High throughput nanofabrication of silicon nanowire and carbon nanotube tips on AFM probes by stencil-deposited catalysts. *Nano Lett.* **11**, 1568–1574 (2011). 21446752 10.1021/nl104384b

[30] Yazdanpanah, M. M. et al. Micro-wilhelmy and related liquid property measurements using constant-diameter nanoneedle-tipped atomic force microscope probes. *Langmuir* **24**, 13753–13764 (2008). 18986184 10.1021/la802820u

[31] Kang, R., Pan, A. & Cui, B. Fabrication of silicon sharp nanocones using dry etch with periodic oxygen plasma shrinking and wet etch. *J. Vac. Sci. Technol. B*. **42**, 033002 (2024).

[32] Mogab, C. J. The loading effect in plasma etching. *J. Electrochem Soc.* **124**, 1262 (1977).

[33] Stoltz, A. J. et al. Macro-loading effects of electron-cyclotron resonance etched II-VI materials. *J. Electron Mater.* **33**, 684–689 (2004).

[34] Ohara, J., Asami, K., Takeuchi, Y. & Sato, K. Development of RIE-lag reduction technique for Si deep etching using double protection layer method. *IEEJ Trans. Electr. Electron. Eng.* **5**, 125–130 (2010).

[35] Chou T. K. A., N. K. Fabrication of out-of-plane curved surfaces in Si by utilizing RIE lag. *MEMS 2002 IEEE International Conference. Fifteenth IEEE International Conference on Micro Electro Mechanical Systems* 145–148 (2002).

[36] Lai, S. L., Johnson, D. & Westerman, R. Aspect ratio dependent etching lag reduction in deep silicon etch processes. *J. Vac. Sci. Technol. A: Vac., Surf., Films* **24**, 1283–1288 (2006).

[37] Takahata, T., Iwase, E., Matsumoto, K. & Shimoyama, I. Three-dimensional silicon fabrication using microloading effects with a rectangular aperture mask. *J. Micromech. Microeng.* **20**, 075022 (2010).

[38] Hong, C. Y. & Akinwande, A. I. Oxidation sharpening mechanism for silicon tip formation. *Electrochem. Solid-State Lett.* **8**, F13 (2005).

[39] Dey, R. K., Shen, J. & Cui, B. Oxidation sharpening of silicon tips in the atmospheric environment. *J. Vac. Sci. Technol. B, Nanotechnol. Microelectron.: Mater., Process., Meas., Phenom.* **35**, 06GC01 (2017).

[40] Han, X. L., Larrieu, G. & Krzeminski, C. Modelling and engineering of stress based controlled oxidation effects for silicon nanostructure patterning. *Nanotechnology* **24**, 495301 (2013). 24231577 10.1088/0957-4484/24/49/495301

[41] Abbas, Y., Rezk, A., Saadat, I., Nayfeh, A. & Rezeq, M. Photodetection characteristics of gold coated AFM tips and n-silicon substrate nano-Schottky interfaces. *Sci. Rep.* **9**, 18478 (2019). 31537835 10.1038/s41598-019-49908-1 PMC6753060

[42] Amelio, S. et al. Measurements of elastic properties of ultra-thin diamond-like carbon coatings using atomic force acoustic microscopy. *Thin Solid Films* **392**, 7584 (2001).

[43] Phillips, G. N., Siekman, M., Abelmann, L. & Lodder, J. C. High resolution magnetic force microscopy using focused ion beam modified tips. *Appl Phys. Lett.* **81**, 865–867 (2002).

