# Supplementary material for: Batch fabrication of ultra-sharp atomic force microscope probes with stair-shaped handles for high-precision imaging
Source: Microsyst Nanoeng. 2025 Oct 21;11:188. doi: 10.1038/s41378-025-00986-4 (PMC12537982; doi:10.1038/s41378-025-00986-4)
Supplement: Supplementary file 1 — Supplementary Information [file 41378_2025_986_MOESM1_ESM.docx]

**Supplementary Information**

**Commercial Si AFM probes**

The batch fabrication steps for standard and commercial all-Si AFM probes are depicted in Fig. SI-1, and this batch fabrication process is provided by NanoWorld^1^. Initially, a double-side polished <100> Si wafer is utilized (Fig. SI-1a), which undergoes cleaning with acetone, isopropanol (IPA), and a deionized water rinse. Thermal oxidation of both sides of the wafer results in forming SiO_2_ layers (Fig. SI-1b), enhancing selectivity to etching solutions in subsequent steps. Then, a layer of photoresist is spin-coated on the backside of the wafer, followed by photolithography (Fig. SI-1c) and development (Fig. SI-1d) to define the tip patterns. Similarly, a new round of resist spin-coating, baking, photolithography, and development is carried out on the front side of the wafer to define the cantilever patterns (Fig. SI-1e and SI-1f). The pattern transferred from the photoresist to the SiO_2_ layer is done by wet isotropic etching, commonly immersed in hydrofluoric acid (HF) or buffered oxide etchant (BOE) (Fig. SI-1g). The remaining photoresist is then removed by immersing the sample in acetone and using ultrasonication to assist the resist strip (Fig. SI-1h). The successful transfer of the mask to the oxide layer and the adequate resist removal are crucial to prevent micro-masking in subsequent wet anisotropic etching of Si.

Subsequently, anisotropic Si etching using potassium hydroxide (KOH) is performed until the oxide mask at the apex of the pyramid falls off (Fig. SI-1i). Further Si etching needs to be done on the backside of the wafer to obtain a cantilever with the desired thickness and release the AFM probe. However, the newly formed tip needs protection from subsequent KOH etching. For this reason, the front side of the sample is coated by the Si_3_N_4_ protective layer (Fig. SI-1j). Anisotropic wet etching is done on the backside until the desired cantilever thickness is achieved (Fig. SI-1k). Finally, the remaining Si_3_N_4_ is removed in an HF/BOE solution to expose the AFM probes (Fig. SI-1l). Notably, the handle is omitted from the fabrication steps for clarity.


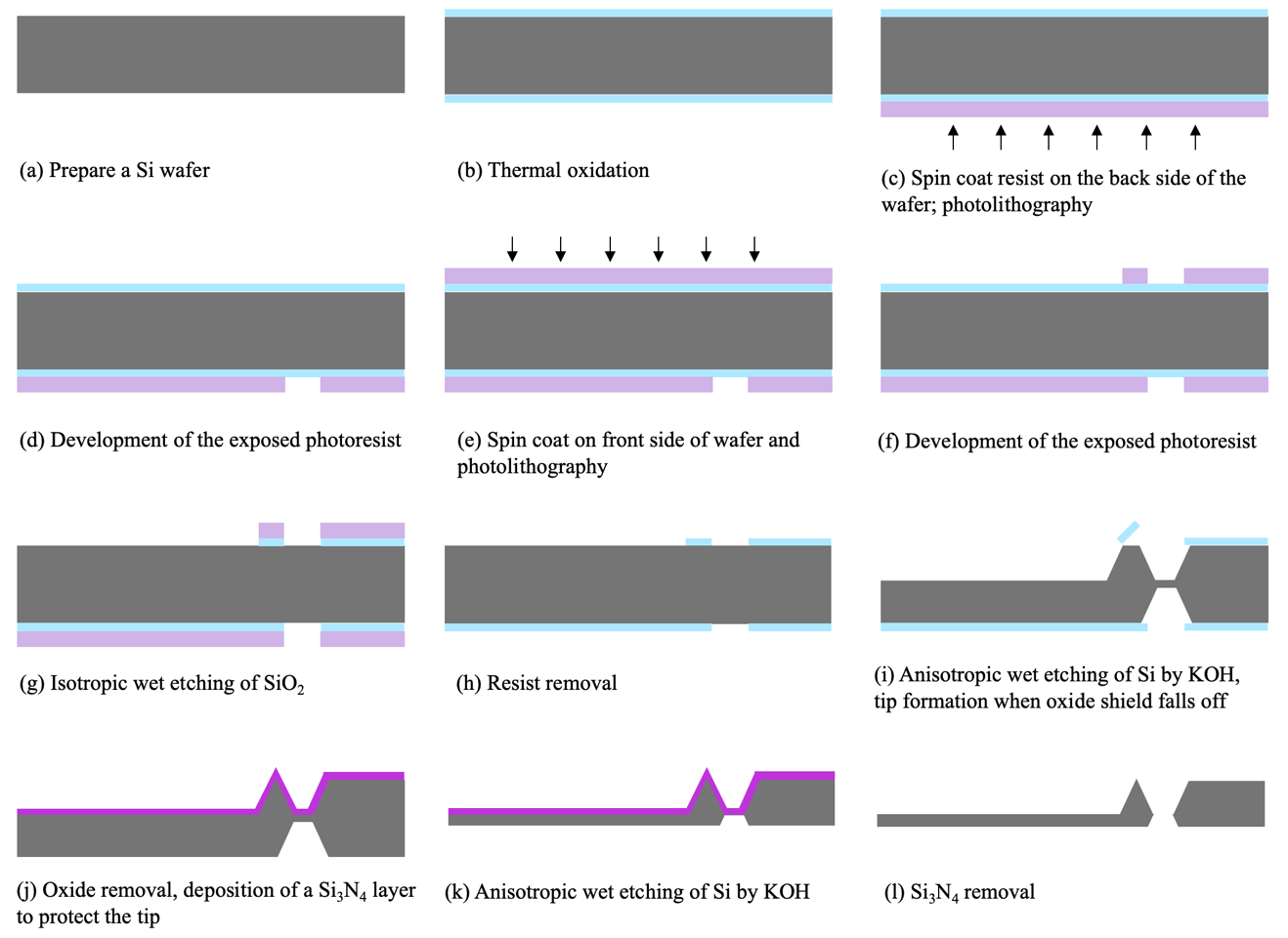


Figure SI-1. Batch fabrication of commercial all-Si AFM probes.

Fig. SI-2 illustrates the profile of the standard tapping probe (Non-contact/Tapping mode, high resonance frequency, NanoWorld Pointprobe NCH)^1^. This pyramidal probe features a resonance frequency ranging from 250 to 390 kHz, coupled with a force constant spanning from 21 to 78 N/m. Its dimensions include a cantilever length of 120 to 130 μm, a width ranging from 25 to 35 μm, and a thickness of 3.5 to 4.5 μm.


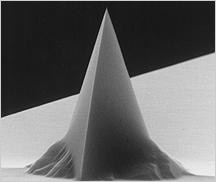


Figure SI-2. The profile of the standard tapping probe, and the probe type is NanoWorld Pointprobe NCH.

**Equations employed in the MATLAB simulation**

Based on the displacement of the cantilever’s unfixed end in the vertical direction for rectangular cantilevers, the spring constant (also termed as force constant) *k* and the resonance frequency *f* are calculated by the following equations:

$$\begin{aligned} k= \frac{Ewt^{3}}{4l^{3}}\#\left( 1 \right) \end{aligned}$$

$$\begin{aligned} f= \frac{1}{2\pi} \sqrt{\frac{k}{m_{0}}}=0.162\sqrt{\frac{E}{\rho}}\frac{t}{l^{2}}\#\left( 2 \right) \end{aligned}$$

*E* is Young’s modulus of the cantilever material, *w* is the cantilever width, *t* is the thickness, and *l* is the length. *ρ* is the density, and m_0_ is the effective mass of the cantilever. As can be seen, the resonance frequency is inversely proportional to the square of the length and proportional to the thickness. An increase in length decreases the frequency very fast; therefore, the resonance frequency of the tip is mainly determined by the length. The spring constant is influenced by its width, the cube of its thickness, and is inversely proportional to the cube of its length. Given the variability in these dimensions, determining the exact spring constant values will be complex. To address this, a MATLAB 4D plot can be employed to identify a set of dimensions that yield a desired spring constant. Once appropriate dimensions are determined, the resonant frequency can be calculated using these specific values.

**Simulation-based analysis to determine the dimensions**

For Si, *E* is 170 GPa, and *ρ* is 2329 kg/m^3^. Previous studies and commercially used AFM probes for tapping mode suggest that the cantilever length ranges from 80 μm to 150 μm, the width ranges from 0 μm to 40 μm, and the thickness ranges from 0.5 μm to 5 μm. Considering this analysis, a MATLAB simulation was conducted to determine the spring constants for the all-Si probe, as illustrated in Fig. SI-3.


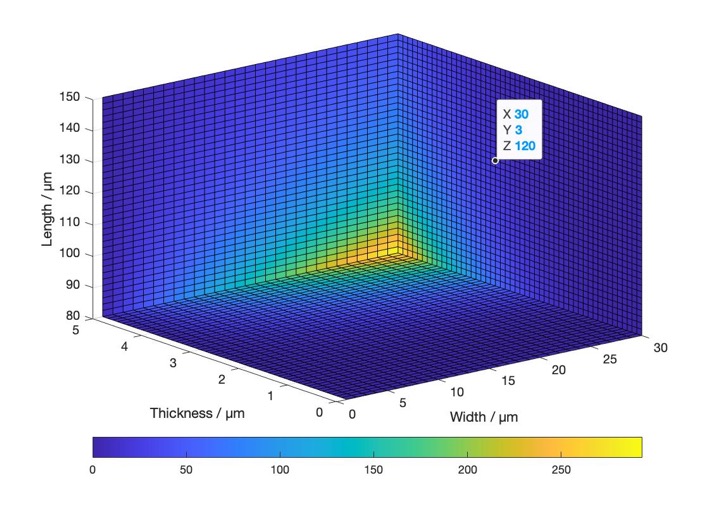


Figure SI-3. The 4D plot of spring constant k (unit N/m) and dimensions w,t,l.

As shown in the 4D slice diagrams, a thick and short cantilever exhibits a high spring constant, making it suitable for non-contact mode AFM operations (otherwise, for contact mode, it is stiff and can damage the sample). Conversely, a thin and long cantilever has a low spring constant, rendering it appropriate for contact mode AFM operations, as it is soft and does less damage during scanning. Cantilevers with moderate spring constants and frequencies can be utilized for AFM tapping mode, offering a delicate balance between scanning speed and image resolution. The 4D plot theoretically allows for any combination of dimensions, resulting in a spring constant falling within the range of 0.5-50 N/m for the cantilever. However, in Si fabrication, cantilevers with a thickness below 2.5 μm may tend to curl due to stress post-release, while a width smaller than 5 μm is hard to align for photolithography and laser beam detection. Thus, an initial setting of the cantilever width at 30 μm (the width exhibits no influence on the spring constant and resonance frequency) and thickness at 3 μm is proposed. Lengths ranging from 80 to 150 μm can be achieved during the fabrication, prompting the tentative selection of a length of 120 μm. Setting the dimensions as (*w, t, l*) = (30, 3, 120) yields a spring constant of 19.92 N/m and a resonance frequency calculated at 288.35 kHz.

The fundamental concept behind the finite element method involves dividing a constructed model into smaller elements and solving the resulting differential equations in the frequency domain. This method enables the creation of accurate models that can be progressively computed following arbitrary partitioning. The benefit of this approach is its ability to accurately simulate complex structures, particularly when the mesh partitioning is finely tuned. However, this precision comes due to increased computational workload and slower simulation speeds, necessitating more powerful computing resources. Consequently, the finite element method is best suited for accurately calculating moderately sized complex structures. Notably, commercial simulation software like COMSOL is built upon the principles of the finite element method.

In COMSOL Multiphysics, a geometric model of the cantilever was created with dimensions specified as follows: 120 μm in length, 30 μm in width, and 3 μm in thickness (the overall shape of the AFM cantilever resembles an inverted T-shaped pattern with a small trapezoid on the free end). We fix one end of the cantilever beam to simulate its connection to the substrate while leaving the other end free to represent the absence of external constraints. Then, we apply an external force (F(t)=F_0_sin(ωt)) at the free end to obtain the vibration response of the cantilever, thereby enabling the simulation of its resonance frequency (303.75 kHz). When a constant external force is applied to the free end, the cantilever undergoes elastic deformation, with the magnitude of deflection proportional to the applied force. The spring constant describes the linear correlation between this deflection and the external force, measuring the cantilever's stiffness and elasticity properties. As illustrated in Fig. SI-4, the maximum/minimum value of the applied force is 50.62/9.38 μN, and the maximum/minimum value of the cantilever’s displacement is 2.52/0.42 μm. As a result, the simulated spring constant is 19.64 N/m.


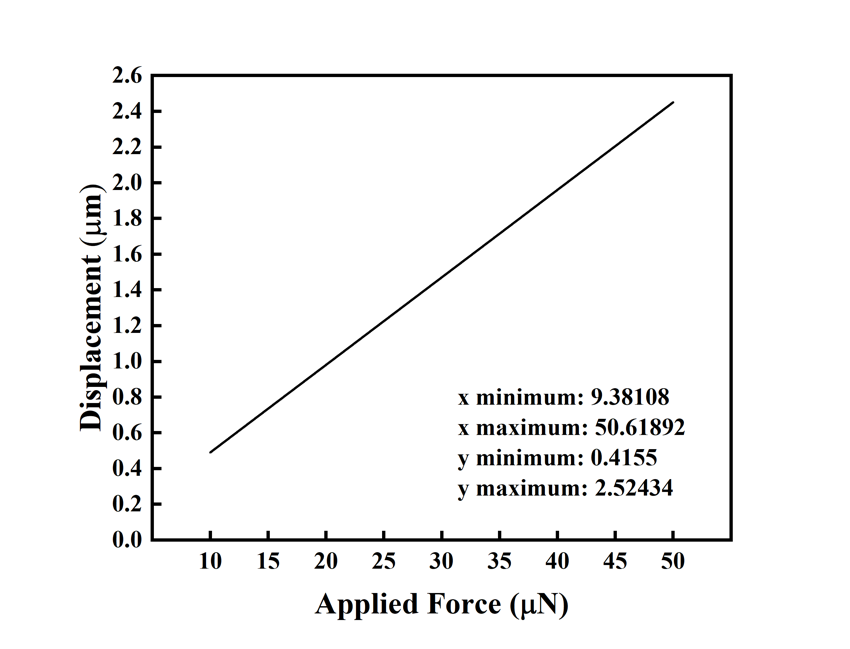


Figure SI-4. The plot of the displacement of a rectangular cantilever with 30 μm in width, 3 μm in thickness and 120 μm in length.

**Product dimensions and profiles**


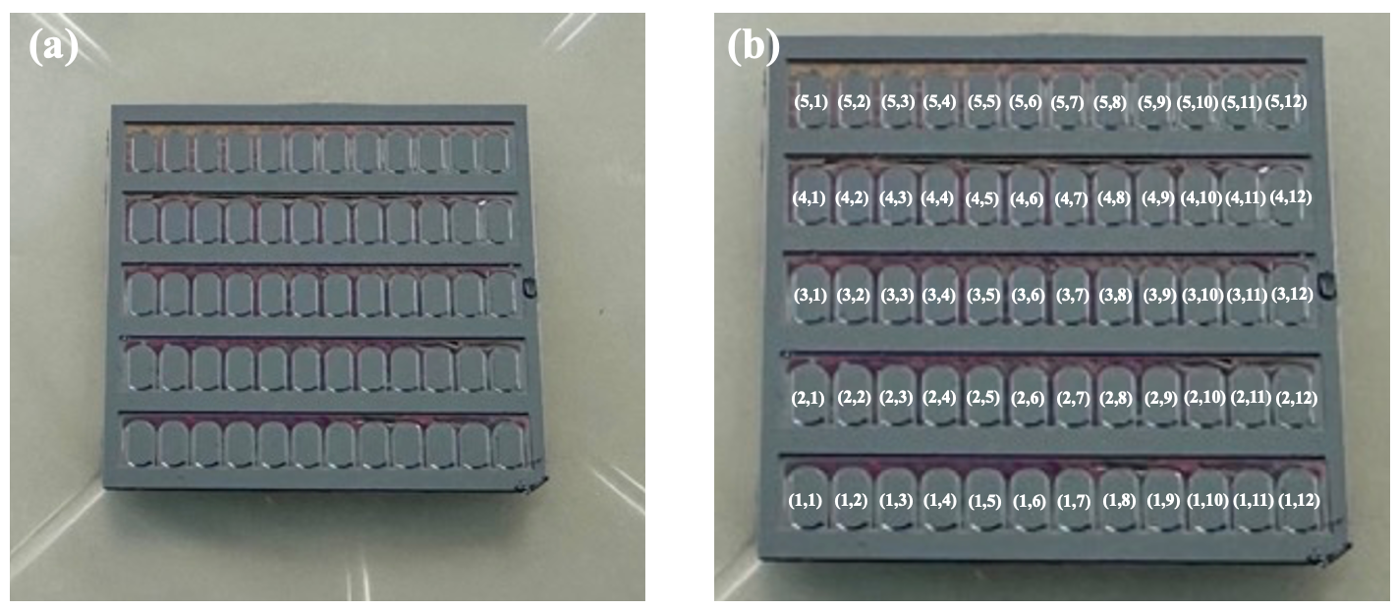


Figure SI-5. (a) The actual product of AFM probes with dimensions of 5 cm × 5 cm, and (b) Coordinate indexing of each individual probe for identification and tracking.

Table SI-1. Dimensions of 46 fabricated Si HAR AFM probes, including tip diameters and cantilever lengths. Note: The average tip diameter is 10.54 ± 0.88 nm.


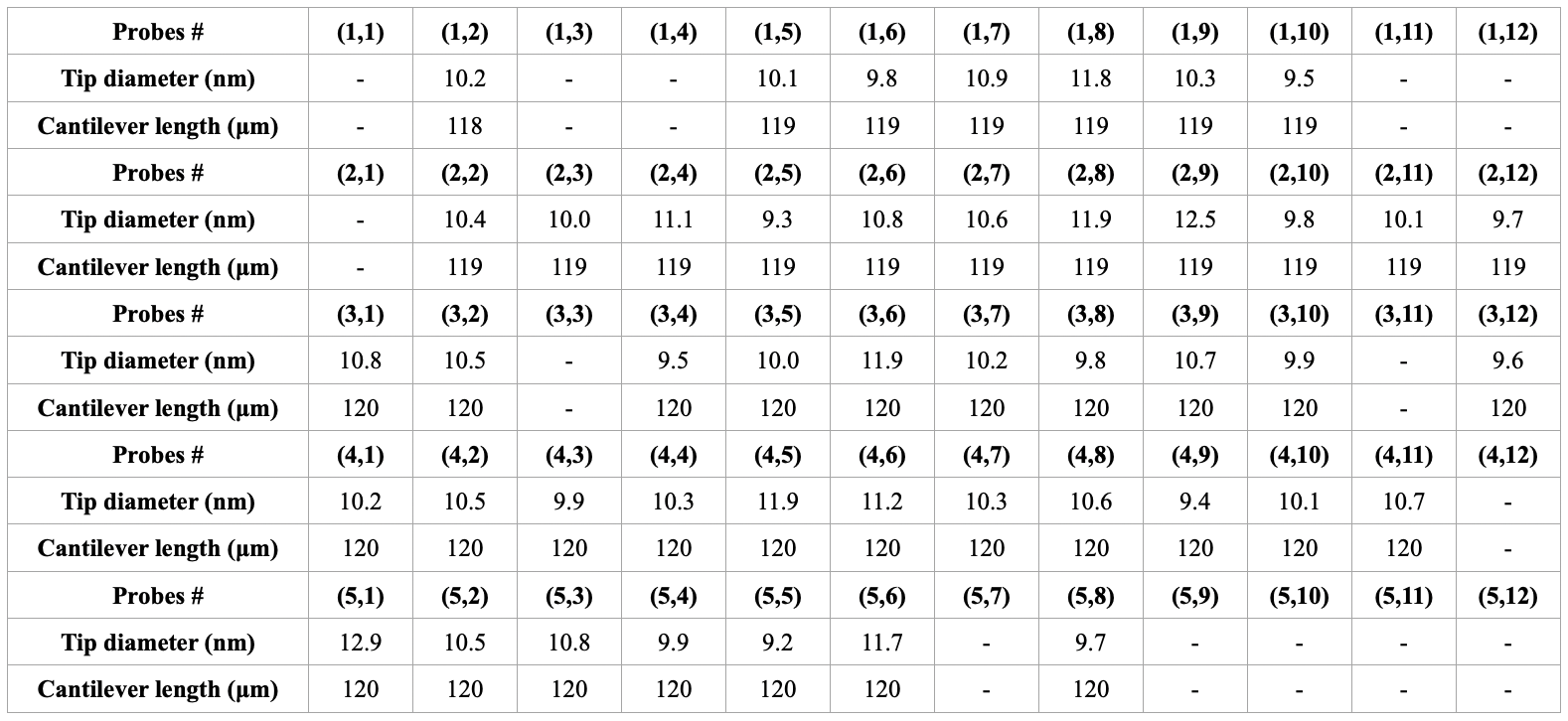


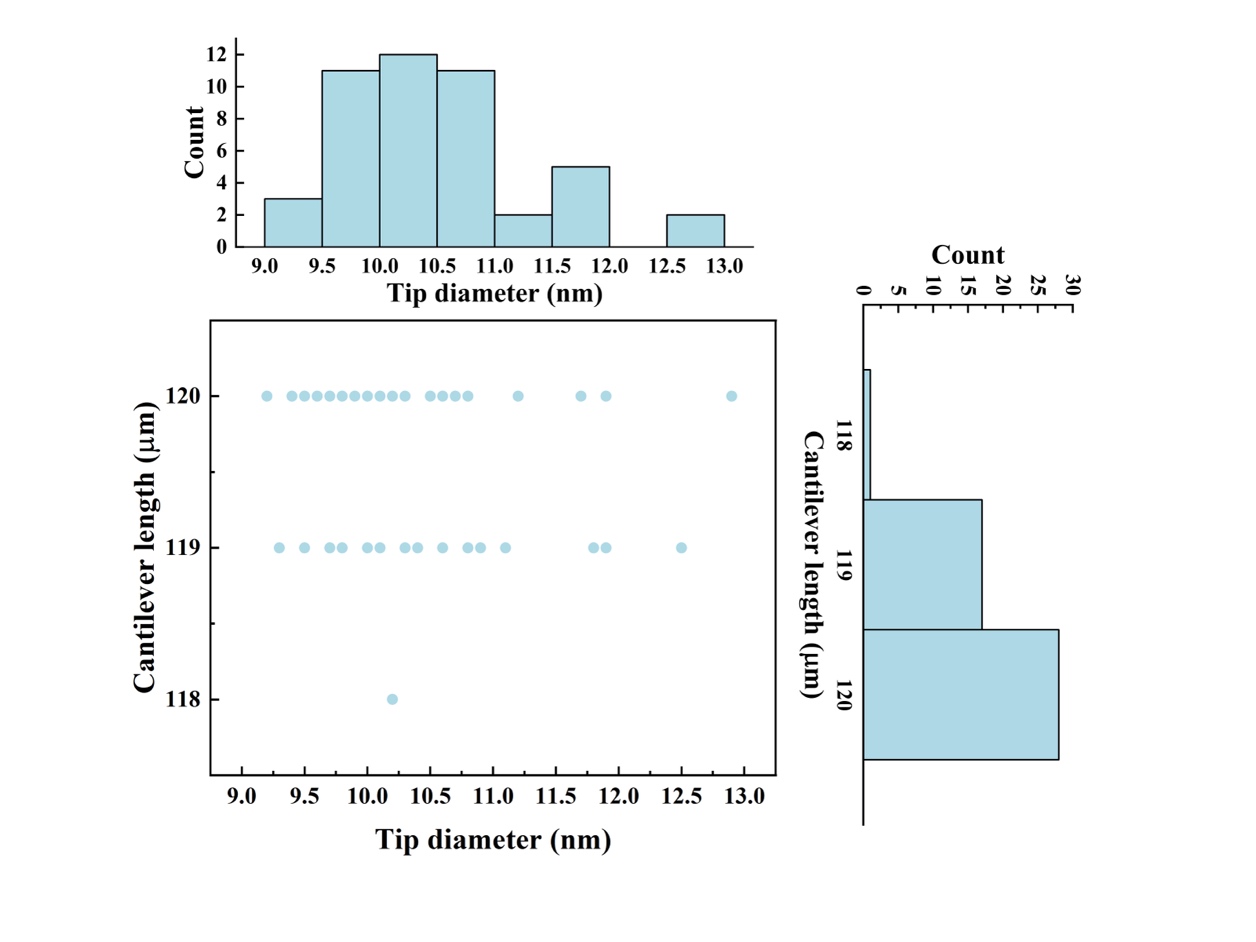


Figure SI-6. Scatter plot and box plot of the dimensions of 46 fabricated Si HAR AFM probes, showing the distribution of tip diameters and cantilever lengths.

**Pattern design in handle etching**

In the test experiment, a 5 cm × 5 cm Si piece is cleaned in acetone, IPA and 2-minute O_2_ plasma. Then, a layer of positive photoresist AZ4620 is spin-coated and soft-baked at 110 ℃ for 90 seconds. The resist is exposed by MLA and then developed. Next, Si is etched by 800 cycles standard Bosch process that gives an overall etching depth of around 420 μm for an open area. The cross-sectional profile after 250 cycles of the standard Bosch process is depicted in Fig. SI-7a, showing varying etching depths across different areas. The vertical sidewall confirms the suitability of the Bosch recipe for this design. Subsequently, 550 additional Bosch cycles were performed, followed by a 20-minute isotropic etching using an SF_6_-only recipe. This process further etched the Si down to approximately 260 μm and laterally "flattened" the steps. The final handle profile, shown in Fig. SI-7b, reveals etched depths of 71 μm, 151 μm, 168 μm, and 287 μm for the major steps, respectively. Although the step heights are unevenly distributed, they can be effectively utilized in AFM probes to minimize the potential for laser light blocking by the final step adjacent to the cantilever.


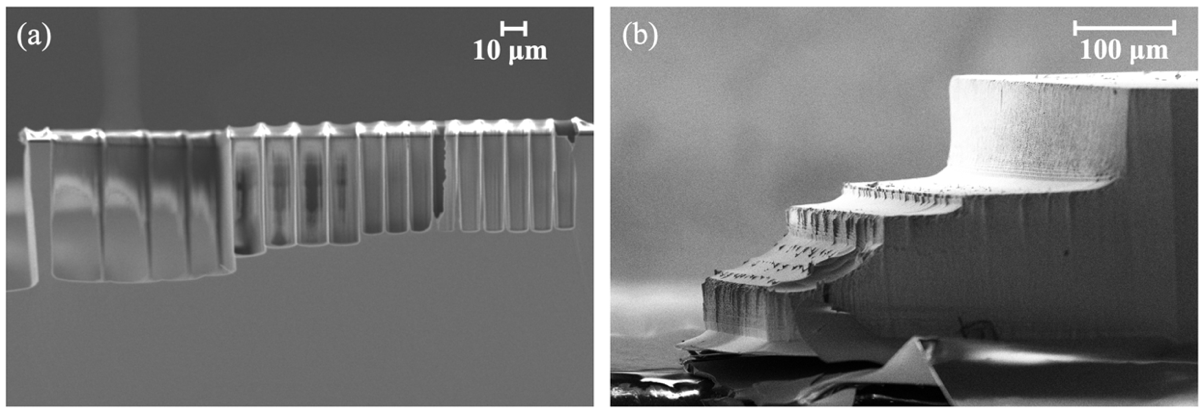


Figure SI-7. The cross-sectional profile of the handle part after (a) 250 cycles of standard anisotropic Bosch etching, (b) 550 additional cycles of standard anisotropic Bosch etching, followed by SF_6_ isotropic etching and 50 more cycles of Bosch etching.

**Scanning lifetime/durability**


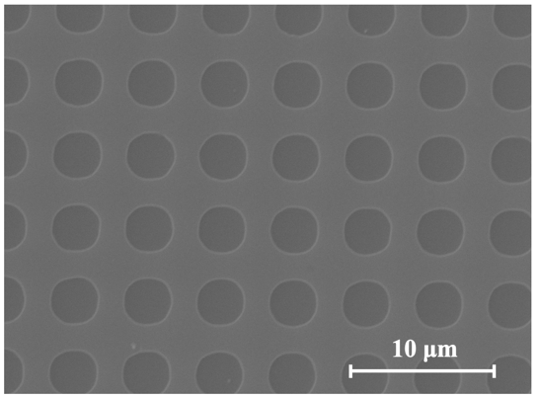


Figure SI-8. SEM image of the standard scanning sample features a hole array with a 2.5 µm diameter, a 5 µm period, and a depth of 300 nm.

The scanning was performed at a rate of 0.6 Hz over an 18 µm × 18 µm area, with a resolution of 256 pixels per scanning line. After 2 hours of continuous scanning on above sample (Fig. SI-8), the HAR probe maintained excellent image quality, as shown in Fig. SI-9b. Even after 3 hours of scanning (Fig. SI-9c), the image quality remained consistent despite noticeable tip wear, with the apex diameter increasing to approximately 40 nm (Fig. SI-9d). By the end of 8 hours of continuous scanning, the tip apex diameter had expanded to ~100 nm (Fig. SI-9e); however, the scanning quality remained unaffected. This demonstrates that the fabricated probe offers a scanning lifespan of at least 3 hours within the 40 nm precision range and up to 8 hours within the 100 nm precision range. For Si probes, tip wear is primarily attributed to electrochemical corrosion, where the continuous formation of an oxide layer on the Si surface accelerates degradation during extended use.


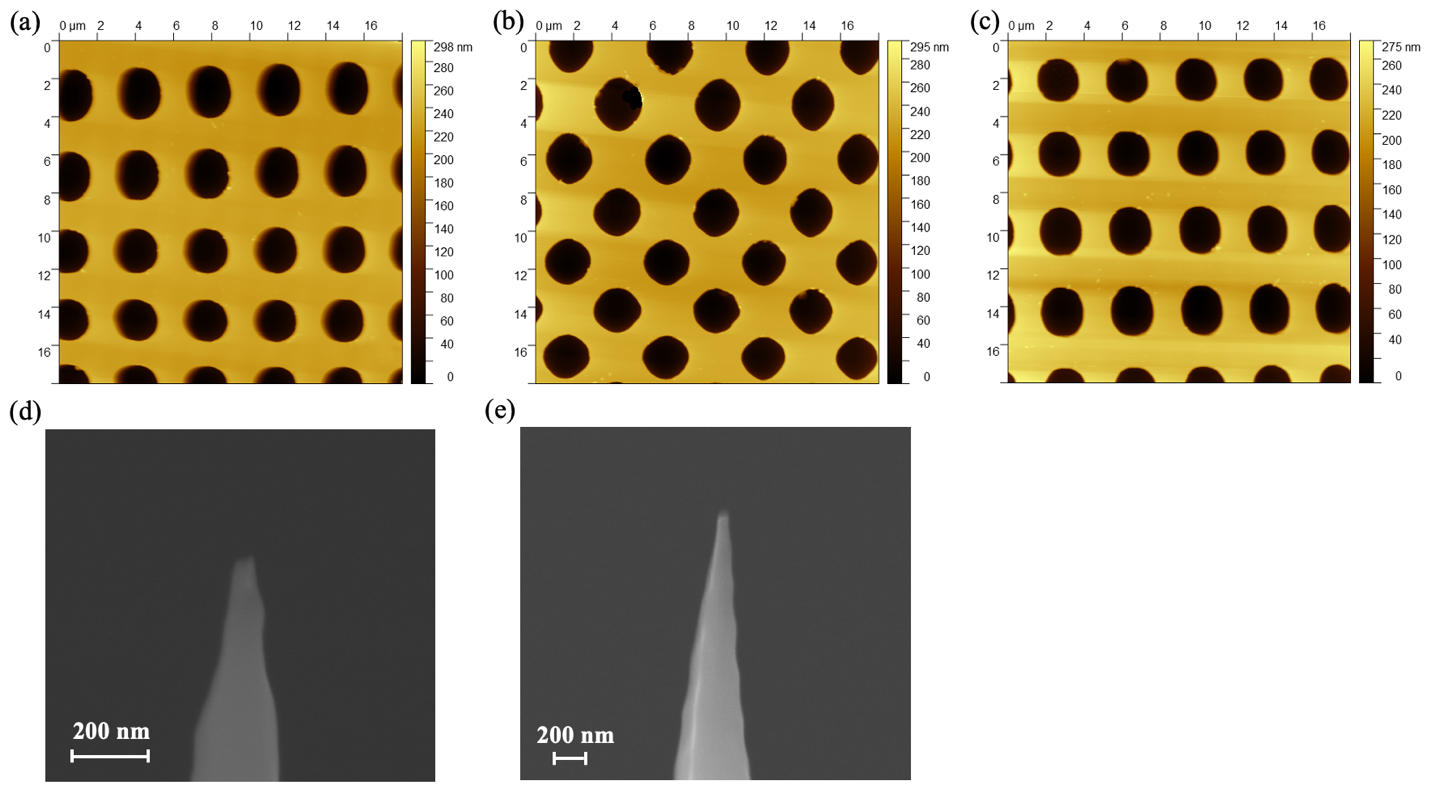


Figure SI-9. The AFM scanning results of (a) the first scan of the fabricated tip, (b) the fabricated tip used for 120 minutes, (c) the fabricated tip used for 180 minutes, (d) the SEM image of the used Si probe after 3-hour continuous scanning, (e) the SEM image of the used Si probe after 6-hour continuous scanning.

**Validation of the repeatability and yield rate**

To verify repeatability and improve the yield rate, we repeated the fabrication process for a second batch of all-Si probes. In this batch, the cantilever length was adjusted to 140 µm to produce probes optimized for imaging soft or fragile samples in AFM tapping mode. The fabrication steps remained identical to those described in the main content. After removing the buried oxide, 50 probes were determined to be intact and usable, exhibiting a probe tip height of 7 µm, a cantilever thickness of 3 µm, a cantilever length of 140 µm, and an overall handle thickness of 300 µm. This indicates a yield rate of approximately 83% for this batch of probes. An intact probe structure is shown in Fig. SI-10a and b, highlighting a tip diameter of 170 nm.


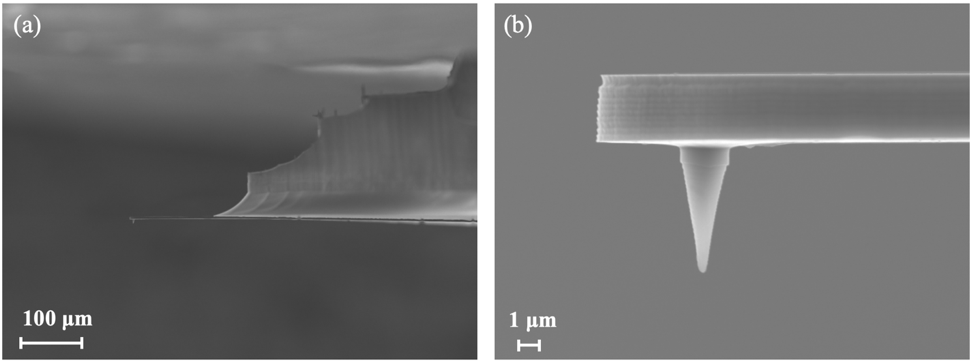


Figure SI-10. SEM images of (a) the side view of the AFM probe with the cantilever length of 140 µm, and (b) the enlarged view of the cantilever and the tip before oxidation sharpening. All images were captured using a JOEL JSM-7200F SEM with the samples mounted on a 90° tilted stage.

After the thermal oxidation at 950 ℃ for 8 hours and the grown SiO_2_ removal with BOE, the final AFM probe product is presented in Fig. SI-11, showcasing a final sharpened Si tip apex measuring approximately 20 nm.


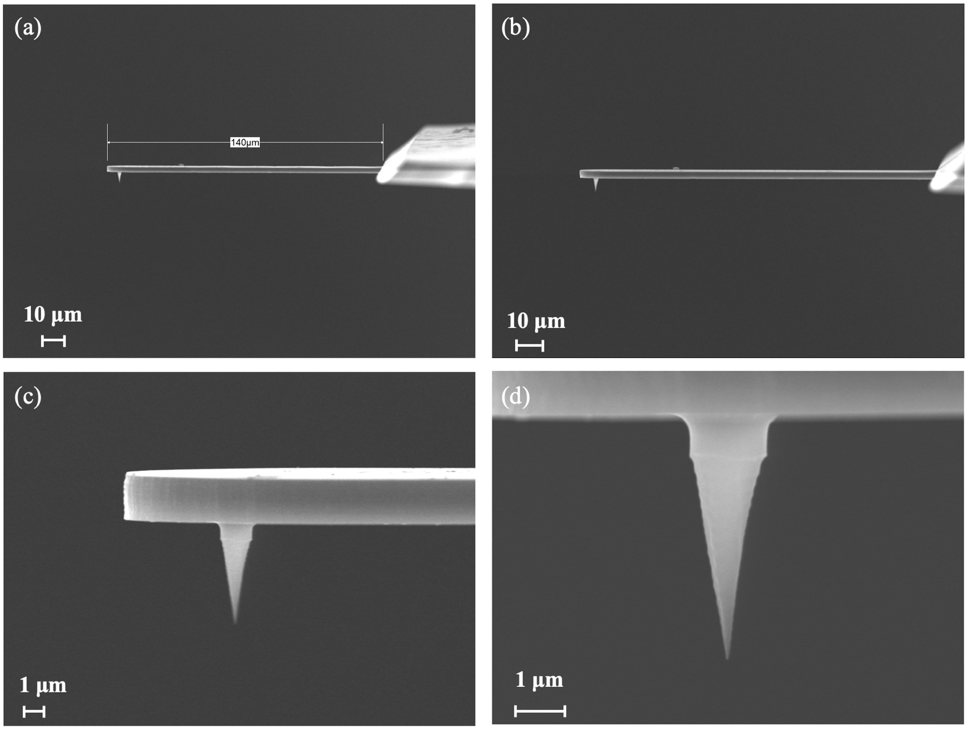


Figure SI-11. SEM images of (a) the final product of the AFM probe with 140 µm cantilever after oxidation sharpening, and (b-d) the enlarged view of the cantilever and the tip. All images were captured using a JOEL JSM-7200F SEM with the samples mounted on a 90° tilted stage.

The fabricated AFM probe was installed on the Aist-NT SmartSPM 1000 in AFM tapping mode, demonstrating that our probes can mounted on various AFM models to showcase their versatility. The resonance frequency of this probe was measured to be 227.26 kHz, as displayed in Fig. SI-12.


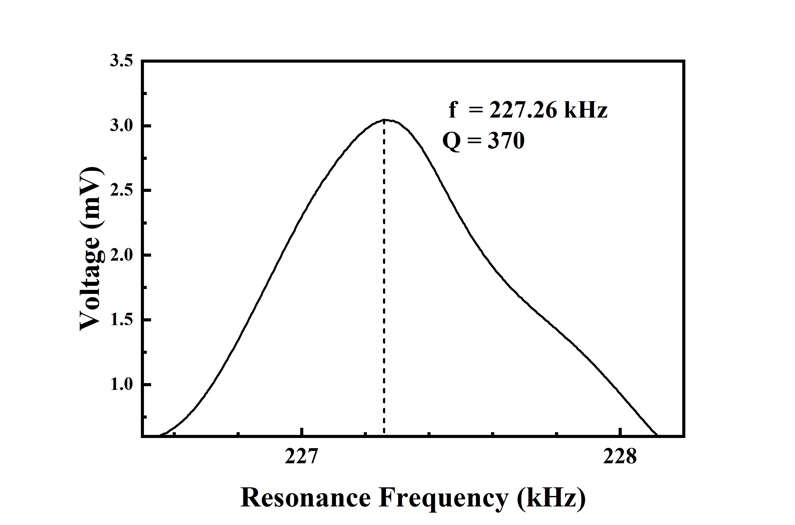


Figure SI-12. Frequency response at resonance of a cantilever, which was mounted on the Aist-NT SmartSPM 1000 in AFM mode.

Here, a soft sample with a photoresist pattern was prepared. Initially, a layer of positive resist AZ3330 was spin-coated onto a bare Si wafer. Subsequently, a line array with a width of 2.5 µm and a period of 5 µm was exposed using an MLA. After development, the final depth reached 3.2 µm, and the profile of the resist line exhibited a positive taper. The resulting scanning image is depicted in Fig. SI-13a and b, with a scan rate of 0.5 Hz (2 µm/s tip velocity). Notably, the final height precisely matches the patterned dimensions. However, when attempting to use the commercial AppNano probe to scan this sample, issues arose because the sample was coated with AZ4620 photoresist, which is highly adhesive. This resulted in the commercial probe experiencing varying degrees of sticking and tip immersion during scanning, preventing the acquisition of usable scanning results.


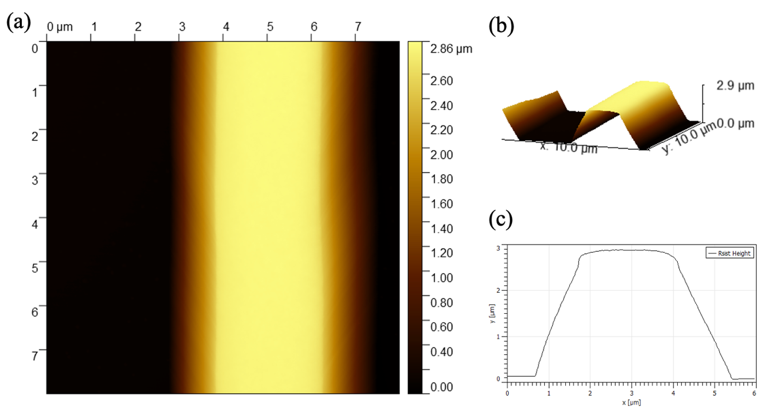


Figure SI-13 (a) The AFM scanning image of the photoresist AZ3330 lines with positively tapered sidewall, (b) the 3D view image, and (c) the cross-sectional view.

**References**

1. Russell, P. & Krause, O. *AFM Probe Manufacturing*. *NanoWorld Innovative Technologies* (2008).
